# Light-To-Moderate Raw Garlic Consumption Frequency Is Inversely Associated With Thickened Carotid Intima-Media Thickness: A Population-Based Study

**DOI:** 10.3389/fnut.2021.648821

**Published:** 2021-03-31

**Authors:** Yunyun Liu, Ge Meng, Qing Zhang, Li Liu, Hongmei Wu, Yeqing Gu, Shunming Zhang, Tingjing Zhang, Xuena Wang, Shaomei Sun, Ming Zhou, Qiyu Jia, Kun Song, Fengling Tan, Kaijun Niu

**Affiliations:** ^1^The Second Affiliated Hospital of Soochow University, Suzhou, China; ^2^Nutritional Epidemiology Institute and School of Public Health, Tianjin Medical University, Tianjin, China; ^3^Department of Toxicology and Sanitary Chemistry, School of Public Health, Tianjin Medical University, Tianjin, China; ^4^Health Management Center, Tianjin Medical University General Hospital, Tianjin, China; ^5^Tianjin Key Laboratory of Environment, Nutrition and Public Health, Tianjin, China

**Keywords:** cross-sectional study, logistic regression analysis, epidemiology, dietary raw garlic consumption, thickened carotid intima-media thickness

## Abstract

**Background:** Previous animal and clinical studies have reported beneficial effects of garlic preparations on carotid intima-media thickness (cIMT). However, no epidemiological study has yet investigated the association between dietary raw garlic consumption and cIMT in the general population. The objective of this study was investigating the association between dietary raw garlic consumption and thickened cIMT in Chinese adults.

**Methods:** This cross-sectional study used data from the Tianjin Chronic Low-grade Systemic Inflammation and Health Cohort Study. A total of 4,329 general adults from 2015 to 2017 were included in this study. Frequency of consumption of raw garlic was summarized as four categories for analysis: < 1 time/week, 1 time/week, 2-3 times/week, ≥4 times/week with a validated food frequency questionnaire. The thickened cIMT was defined as common carotid artery IMT ≥ 1.0 mm or a carotid bifurcation IMT ≥ 1.2 mm by ultrasonography. Multivariable logistic regression analysis was used to examine the association between frequency of raw garlic consumption and thickened cIMT.

**Results:** The prevalence of thickened cIMT is 22.9% among these participants. The adjusted odds ratios (95% confidence intervals) associated with the different frequencies were 1.00 (reference) for < 1 time/week, 0.74 (0.59, 0.94) for 1 time/week, 0.71 (0.55, 0.92) for 2–3 times/week, and 0.94 (0.71, 1.25) for ≥ 4 times/week.

**Conclusions:** Light-to-moderate raw garlic consumption was inversely associated with thickened cIMT, whereas greater raw garlic consumption (i.e., ≥4 times/week) was not associated with thickened cIMT. Future longitudinal studies should be conducted to test these findings.

## Background

Cardiovascular disease (CVD) is recognized as a global public health issue, causing death and disability worldwide (1). According to World Health Organization's Fact Sheet reports, 17.9 million deaths were caused by CVD in 2016 and such deaths mainly took place in low and middle income countries (2, 3). Carotid intima-media thickness (cIMT) was an effective early marker of atherosclerosis and an independent risk predictor of CVD (4–6). As an ultrasound and non-invasive indicator for detecting preclinical atherosclerosis, cIMT is widely used in observational study and atherosclerosis screening due to its accessibility and convenience. Thickened cIMT has been shown to predict cardiovascular morbidity and mortality (7). Oxidative stress and inflammation are considered to be the main causes of atherosclerosis (8, 9). Foods, as a source of body nutrients, are rich in bioactive phytochemicals and bionutrients that have anti-oxidation and anti-inflammation properties (10). Thus, diet plays a significant role in the development of atherosclerosis (11–14).

Raw garlic is widely consumed in China, particularly in the north of China that is the largest producer and exporter of garlic (15). There are abundant organo-sulfur compounds (OSCs) containing raw garlic, especially allicin, which is well-known for its anti-oxidant and anti-inflammatory properties (16). Therefore, we speculated that consumption of raw garlic may have a beneficial effect on the prevention of thickened cIMT. Most people usually consume the roasted form of garlic due to the pungent odor of raw garlic. However, heating causes alliinase inactivation and blocks subsequent odorous OSCs formation, which is assumed to be associated with the reduction of garlic's bioactivity (17, 18). Several randomized clinical trials have focused on the impact of garlic preparations on cIMT in patients with atherosclerosis, the results are inconsistent (19–21). Given that there is still no available population-based data about the association of raw garlic consumption with cIMT, we conducted this large-scale cross-sectional study to investigate how raw garlic consumption frequency is associated with thickened cIMT among the general adult population.

## Methods

### Study Population

**Figure 1:**
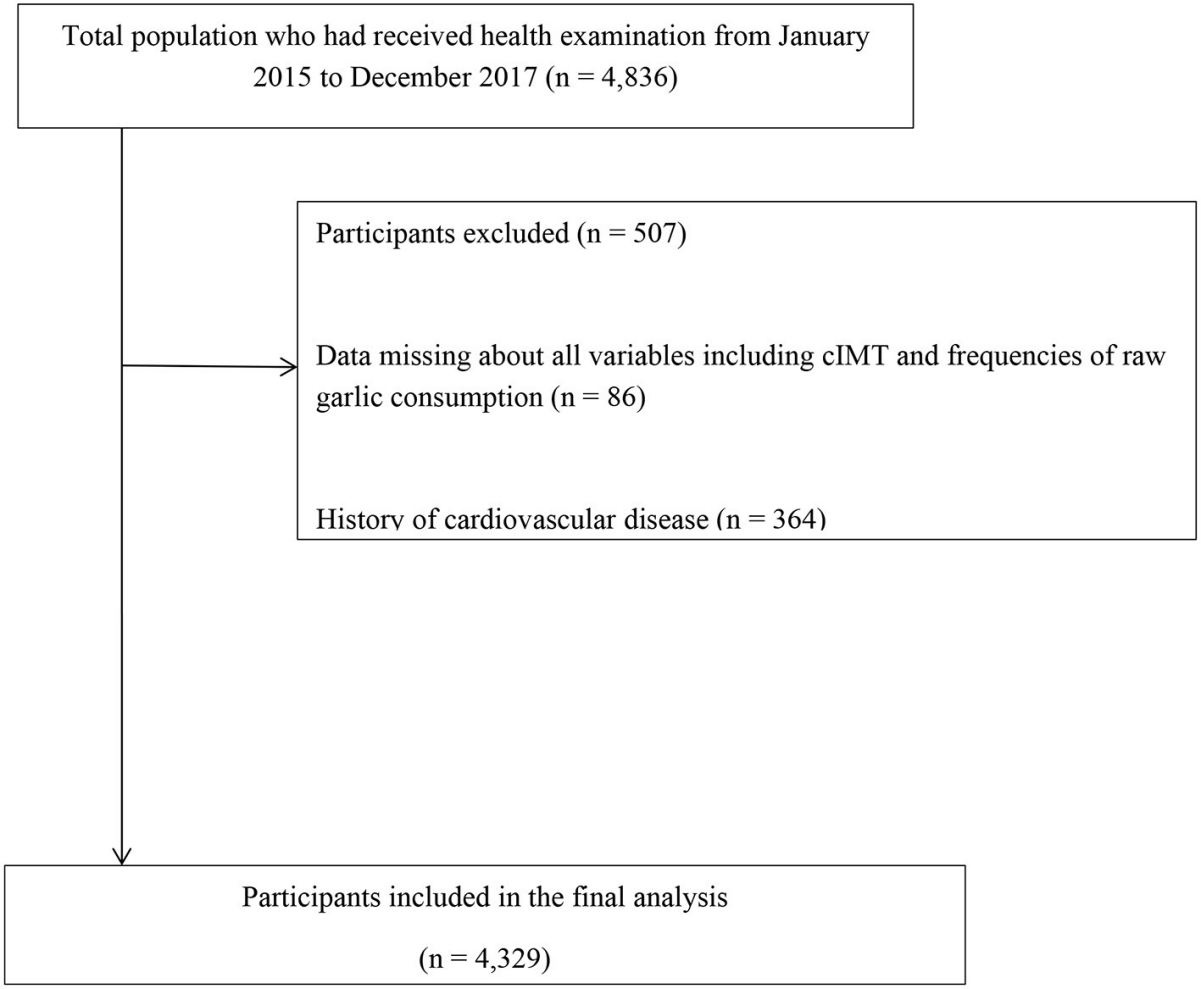
Flow diagram of the participants' selection for the study of association between raw garlic consumption frequency and thickened intima media thickness.

This current research was a population-based study, and the study participants were all from Tianjin Chronic Low-grade Systemic Inflammation and Health (TCLSIH) Cohort Study. TCLSIH is a large prospective dynamic cohort study aimed to explore the association between chronic low-grade systemic inflammation and health status. More details about the TCLSIH Cohort have been published elsewhere (22). The data from 2015 to 2017 was used in this analysis. All participants were recruited when they received their annual health examinations at health management centers and community management centers of Tianjin, China. During their health examination, a total of 4,836 individuals received cIMT test and were asked to answer questionnaires about their lifestyle. After the exclusion of participants who had missing data about all variables (*n* = 86), or who had a history of CVDs (*n* = 364) or cancer (*n* = 57), there were 4,329 participants [mean age ± standard deviation: 50.8 ± 10.5 years; males, 55.7%] in the final cross-sectional study (Figure 1).

### Assessment of Dietary Consumption

The participants were instructed to complete a modified version of the food frequency questionnaire (FFQ) about their diets over the past month, which included 100 items with specified serving sizes. The FFQ included 7 frequencies, ranging from “almost never eat” to “twice or more per day” for foods and 8 frequencies, ranging from “almost never drink” to “twice or more times per day” for beverages. Information on raw garlic consumption was assessed via the simple question: “over the previous one month, how often on average did you consume raw garlic?” Participants selected one of 7 options (almost never, < 1 time/week, 1 time/week, 2–3 times/week, 4–6 times/week, 1 time/day, and ≥2 times/day) for the frequency of raw garlic consumption in the preceding month. In the analysis, the frequency of raw garlic consumption was categorized into four groups: < 1 time/week (reference), 1 time/week, 2–3 times/week, and ≥4 times/week. In the study region, the weight of a specific serving of raw garlic is 9 grams for men and 7 grams for women and average daily nutrients consumption was calculated based on Chinese food composition table (23). The reproducibility and validity of the FFQ have been tested on 150 participants drawn randomly from the cohort using data from repeated measurements of the FFQ ~3 months apart and 4-day weighed diet records (WDRs). The Spearman correlation coefficients between FFQ and WDRs were 0.69 for raw garlic consumption, 0.49 for energy intake, and 0.35–0.54 for nutrients (*n*-3 fatty acid, fat, and carbohydrate). Spearman's rank correlation coefficients between two FFQs were 0.68 for energy intake, 0.62–0.79 for food items (fruits, vegetables, sweet foods, and beverages), and 0.78 for raw garlic, which indicated that the FFQ represented long-term consumption of foods and beverages for participants.

### Assessment of Thickened cIMT

The cIMT was measured by trained and certified sonographers, using iU Elite (Royal Philips) equipped with a L9-3 transducer. All participants, lying in the supine position, were examined with their heads turned 45° to the contralateral side of the artery. Sonographers scanned the far wall of the common carotid artery (CCA) and the carotid bifurcations at both the left and right carotid arteries, measuring the distance from the edge of the first echogenic line to the edge of the second echogenic line. The definition of thickened cIMT is CCA IMT ≥1.0 mm or a carotid bifurcation IMT ≥1.2 mm (24, 25). All these measurements were repeated three times: the intrameasure and intermeasure CVs were < 2.90%.

### Assessment of Other Variables

Blood samples were collected in siliconized vacuum plastic tubes for laboratory tests, including fasting blood glucose (FBG), total cholesterol (TC), triglycerides (TG), low-density lipoprotein cholesterol (LDL-C), and high-density lipoprotein cholesterol (HDL-C). FBG was measured by the glucose oxidase method (26). For lipids, TC and TG were measured by the enzymatic colorimetric method, LDL-C was measured by the olyvinyl sulphuric acid precipitation method, and HDL-C was measured by the chemical precipitation method using appropriate kits on a Cobas 8000 analyzer (Roche, Mannheim, Germany). Height and weight were recorded with a standard protocol. The calculation of body mass index (BMI) was equal to weight (kg)/height (m^2^). Waist circumference (WC) was measured at the umbilical level when participants stood and breathed normally. Blood pressure (BP) was measured twice at the upper right arm using an automatic device (TM-2655P, A&D Company, Ltd., Tokyo, Japan) after 5 min rest in a seated position. The mean value of the two readings was used to analyze. Metabolic syndrome (MS) was defined according to American Heart Association scientific statement of 2009 (27). As to socio-demographic variables, such as age, sex, current medication, family history of disease, individual history of disease, as well as drinking status (defined as “everyday,” “sometime,” “ex-drinker,” and “non-drinker”), smoking (defined as “smoker,” “ex-smoker,” and “non-smoker”), recorded from “yes” or “no” response to relevant questions on a questionnaire. Physical activity (PA) in the recent week was assessed by the short version of International Physical Activity Questionnaire (28). The questionnaire asked about the following activities participants had performed during the last week: walking; moderate activity (household activity or child care); vigorous activity (running, swimming, or other sports activities). Metabolic equivalents (METs) hours per week were calculated by the following formula: hours of walking × days per week with walking × 3.3) + (daily hours of moderate intensity activity × days per week with moderate-intensity activity × 4.0) + (daily hours of vigorous activity × days per week with vigorous activity × 8.0). Total PA levels were assessed by METs hours per week (29). The FFQ was also used to evaluate the total energy intake and factor analysis was used to generate major dietary patterns and factor loadings on food items and beverages (g). Varimax rotation was applied for greater interpretability. Three factors were determined after evaluation of eigenvalues (>1.0) and the scree plot test, which explained 22.1% of the variance in dietary consumption (i.e., 9.47% for factor 1, 6.37% for factor 2, and 6.26% for factor 3). Raw garlic and total onion consumption were not included in the calculation of dietary patterns.

More detailed socio-demographic information about education levels, employment status, household income, marital status, living condition, frequency of visiting friends was obtained from the same questionnaire. Level of education was categorized into 2 categories: < College graduate or ≥ College graduate. Marital status was classified as married or unmarried. Occupation was grouped in: managers, professionals, and others. For household income, 10,000 Yuan per month was regarded as the threshold to divide population. Living condition was defined as living alone or with others. Information about the frequency of visiting friends was asked by the question, “do you often visit your friends and relatives?”

### Statistical Analysis

All statistical analyses were performed by SAS 9.3 edition (SAS Institute Inc., Cary, NC, USA). The thickened cIMT was used as dependent variables, and raw garlic consumption frequency was used as independent variables. We use one-sample Kolmogorov-Smirnov test to assess the normality of distribution of continuous variables. Natural log transformation was used for all the continuous variables to improve the normality of the data. Descriptive data are shown as the geometric means (95% confidence interval, CI) for continuous variables or percentages for categorical variables. The differences in variables between the raw garlic consumption frequencies were examined using analysis of variance for continuous variables or logistic regression analysis for variables of proportion. Logistic regression analysis was used to determine the association between raw garlic consumption frequency and thickened cIMT. In model 1, the analysis was conducted with adjustments for age and sex. In model 2, we additionally adjusted for BMI. In model 3, additional variables were adjusted for the following potential confounders: smoking status, drinking status, educational levels, PA, employment status, incomes, total energy intake, marital status, MS, dietary patterns, and visiting friends. In model 4, we additionally adjusted for total onion intake. In addition, variance inflation factor (VIF) was used to assess multicollinearity among covariates. VIF exceeding 10 was a sign of multicollinearity. In model 4, all VIFs were less than 5, showing no multicollinearity among covariates. Participants with missing data about all variables were excluded. Odds ratios (ORs) and 95% CI were calculated. All *P*-values for linear trends were calculated using the frequency of raw garlic consumption as an ordinal variable and two-tailed *P*-values < 0.05 were defined as statistically significant.

We conducted subgroup analyses stratified by age, sex, BMI, alcohol drinking status, smoking status, education level, household income, marriage and MS. Potential interactions between stratifying variables and raw garlic consumption were assessed by adding cross-product terms to the Logistic models. To examine the robustness of the results, we conducted sensitivity analysis by adding in the association analysis three different models for the three dietary patterns adjustments separately.

## Results

The current research included 4,329 participants. We had all participants cIMT measured and a total of 22.9 % of participants had thickened cIMT. Tables 1, 2 shows the general characteristics and dietary factors of participants according to the frequency of raw garlic consumption. Compared with those in the lower frequency group, participants with the higher frequency of raw garlic consumption were older, and were more likely to have higher BMI, WC, TG, SBP, BDP, FBG, and PA, higher “Health” dietary pattern score, “animal foods” dietary pattern score, higher total energy intake and onion intake, but lower HDL level (*P* for all trend < 0.01). Also, they were more likely to have MS, visited friends frequently, or to be male, current smokers, ex-smokers, everyday drinkers, sometimes drinkers, or be married (*P* for all trend ≤ 0.01). In addition, compared with those in the lower frequency group, participants with higher frequency of raw garlic consumption were less likely to live alone, engage in other occupations, or to be non-smokers, non-drinkers (*P* for all trend < 0.01). Otherwise, no statistically significant difference was observed among other variables across garlic consumption groups.

**Table 1:** General characteristics of 4,329 Chinese adults according to the frequency of garlic consumption, the Tianjin Chronic Low-grade Systemic Inflammation and Health Cohort Study 2015–2017.

**Characteristics**	**Frequency of raw garlic consumption**	***P* for trend^a^**			
	**<1 time/week**	**1 time/week**	**2–3 times/week**	**≥4 times/week**	
No. of participants	1,073	1,534	1,032	690	-
Sex (male, %)	42.7	57.6	62.9	61.2	<0.0001
Age (years)	48.5 (47.9, 49.1)^b^	48.8 (48.3, 49.4)	50.9 (50.2, 51.5)	51.5 (50.7, 52.4)	<0.0001
BMI^c^ (kg/m^2^)	24.5 (24.3, 24.7)	25.2 (25, 25.3)	25.6 (25.4, 25.8)	25.4 (25.2, 25.7)	<0.0001
WC^c^ (cm)	83.5 (82.9, 84.1)	85.9 (85.3, 86.4)	87.6 (87.0, 88.3)	87.1 (86.3, 87.8)	<0.0001
TC^c^(mg/dL^d^)	192.5 (190.6, 194.8)	193.7 (191.8, 195.2)	194.1 (191.8, 196.0)	194.5 (191.8,197.2)	0.42
TG^c^ (mg/dL^e^)	112.5 (109.0, 117.0)	124.0 (120.5, 127.6)	132.0 (126.7, 136.4)	126.7 (121.4, 132.0)	<0.0001
LDL-C^b^ (mg/dL^d^)	112.5 (110.6, 114.8)	112.9 (111.3, 114.4)	113.3 (111.0, 115.2)	114.0 (111.7, 116.8)	0.76
HDL-C^b^ (mg/dL^d^)	51.8 (51.0, 53.0)	50.3 (49.5, 51.0)	49.1 (48.3, 49.9)	49.9 (48.7, 51.0)	<0.0001
SBP^c^ (mmHg)	121.5 (120.49, 122.51)	122.3 (121.5, 123.2)	125.2 (124.2, 126.3)	126.5 (125.2, 127.8)	<0.0001
DBP^c^ (mmHg)	76.6 (75.9, 77.3)	77.9 (77.3, 78.5)	79.9 (79.2, 80.6)	79.9 (79.1, 80.8)	<0.0001
FBG^c^ (mg/dL^f^)	92.3 (91.4, 93.4)	93.4 (92.5, 94.1)	94.7 (93.6, 95.8)	95.2 (94.0, 96.5)	<0.01
Physical activity (METs^c^ × hour/week)	9.34 (8.58, 10.2)	9.59 (8.94, 10.3)	12.1 (11.1, 13.2)	12.3 (11.1, 13.7)	<0.0001
Metabolic syndrome (yes, %)	29.9	36.0	44.1	40.9	<0.0001
Smoking status (%)					
Smoker	18.7	25.1	27.4	27.4	<0.0001
Ex-smoker	6.77	7.77	11.4	10.1	<0.001
Non-smoker	74.5	67.1	61.2	62.5	<0.0001
Drinker status (%)					
Everyday	4.82	7.96	11.6	13.8	<0.0001
Sometime	48.6	57.2	58.8	54.9	<0.01
Ex-drinker	11.3	7.63	7.48	8.54	0.02
Non-drinker	35.3	27.2	22.2	22.7	<0.0001
Education level (≥ College graduate, %)	41.2	52.1	45.6	41.3	0.59
Marital status (married, %)	95.0	97.7	98.6	98.4	<0.0001
Living alone (yes, %)	8.56	4.93	4.79	5.01	<0.01
Employment status (%)					
Managers	30.7	40.2	37.5	36.6	0.03
Professionals	10.8	13.0	13.0	12.4	0.29
Other	58.4	46.8	49.5	50.9	<0.01
Household income (≥10,000 Yuan, %)	43.7	48.1	44.3	42.7	0.40
Visiting friends (yes, %)^g^	62.5	64.4	68.4	71.8	<0.0001

a *Analysis of variance or logistic regression analysis*.

b *Geometric mean (95% confidence interval) (all such values)*.

c *BMI, body mass index; WC, waist circumference; TC, total cholesterol; TG, triglycerides; LDL-C, low-density lipoprotein cholesterol; HDL-C, high-density lipoprotein cholesterol; SBP, systolic blood pressure; DBP, diastolic blood pressure; FBG, fasting blood glucose; METs, metabolic equivalents*.

d *To convert mg/dL cholesterol to mmol/L, multiply mg/dL by 0.0259. To convert mmol/L cholesterol to mg/dL, multiply mmol/L by 38.7. Cholesterol of 192 mg/dL = 4.97 mmol/L*.

e *To convert mg/dL triglycerides to mmol/L, multiply mg/dL by 0.0113. To convert mmol/L triglycerides to mg/dL, multiply mmol/L by 88.5. Triglycerides of 124 mg/dL = 1.40 mmol/L*.

f *To convert mg/dL fasting blood glucose to mmol/L, multiply mg/dL by 0.0556. To convert mmol/L fasting blood glucose to mg/dL, multiply mmol/L by 18.0. Fasting blood glucoses of 92 mg/dL = 5.12 mmol/L*.

g *“yes” indicated participants often visit their friends and relatives*.

**Table 2:** Dietary characteristics of 4,329 Chinese adults according to the frequency of garlic consumption, the Tianjin Chronic Low-grade Systemic Inflammation and Health Cohort Study 2015–2017.

**Dietary factors**	**Frequency of raw garlic consumption**				***P* for trend^a^**
	**<1 time/week**	**1 time/week**	**2–3 times/week**	**≥4 times/week**	
No. of participants	1,073	1,534	1,032	690	-
Total energy intake (kcal/day)	1853.5 (1822.5, 1885.0)^b^	1932.3 (1905.2, 1959.7)	2081.3 (2045.9, 2117.4)	2167.3 (2122.2, 2213.3)	<0.0001
Onion intake (g/d)	4.69 (4.19,5.19)	5.08 (4.66,5.49)	7.79 (7.28,8.3)	11.9 (11.27, 12.52)	<0.0001
“Health” dietary pattern score	−0.23 (−0.29, −0.18)	−0.30 (−0.35, −0.26)	0.12 (0.07, 0.18)	0.85 (0.78, 0.92)	<0.0001
“Sweets” dietary pattern score	−0.04 (−0.10, 0.02)	0.02 (−0.03,0.07)	−0.02 (−0.08, 0.04)	0.04 (−0.03, 0.12)	0.72
“Animal foods” dietary pattern score	−0.17 (−0.23, −0.11)	−0.04 (−0.09, 0.01)	0.12 (0.06, 0.18)	0.18 (0.11, 0.26)	<0.0001

a *Analysis of variance or logistic regression analysis*.

b *Geometric mean (95% confidence interval) (all such values)*.

Factors were named descriptively according to the food items with a factor loading >|0.30| with respect to each dietary pattern as follows: “health” dietary pattern (factor 1), “sweets” pattern (factor 2), and “animal foods” pattern (factor 3). The detailed information is available in Supplementary Table 1.

Table 3 shows the adjusted associations between frequency of raw garlic consumption and thickened cIMT. In the model 1, age-, sex-adjusted ORs (95% CI) across raw garlic consumption frequency were 1.00 (reference) for < 1 time/week, 0.82 (0.65, 1.03) for 1 time/week, 0.85 (0.67, 1.08) for 2–3 times/week, and 1.06 (0.82, 1.37) for ≥4 times/week. In the model 2, age-, sex-, and BMI-adjusted ORs (95% CI) of thickened cIMT across the increasing frequency of raw garlic consumption were 1.00 (reference), 0.78 (0.62, 0.98), 0.79 (0.62, 1.01), and 1.02 (0.79, 1.33). In the model 3, adjusted ORs (95% CI) of thickened cIMT across the increasing frequency of raw garlic consumption were 1.00 (reference), 0.75 (0.60, 0.95), 0.73 (0.57, 0.94), and 0.98 (0.74, 1.30). In final model, adjusted ORs (95% CI) of thickened cIMT across the increasing frequency of raw garlic consumption were 1.00 (reference), 0.74 (0.59, 0.94), 0.71 (0.55, 0.92), and 0.94 (0.71, 1.25). Compared with participants who consumed raw garlic < 1 time/week, there were 26 and 29% reduction in the risk of thickened cIMT among people who consumed raw garlic 1 time/week, and 2–3 times/week, respectively. No statistically significant difference was found, when people consumed raw garlic ≥4 times/week.

**Table 3:** Multivariate adjusted odds ratios for thickened cIMT ^a^ according to frequency of raw garlic consumption among Chinese adults, the Tianjin Chronic Low-grade Systemic Inflammation and Health Cohort Study.

**Logistic regression models**	**Frequency of raw garlic consumption**			
	**<1 time/week**	**1 time/week**	**2–3 times/week**	**≥4 times/week**
No. of participants	1,073	1,534	1,032	690
No. of thickened IMT^a^	237	309	250	196
Model 1^b^	1.00 (reference)	0.82 (0.65, 1.03)^c^	0.85 (0.67, 1.08)	1.06 (0.82, 1.37)
Model 2^d^	1.00 (reference)	0.78 (0.62, 0.98)	0.79 (0.62, 1.01)	1.02 (0.79, 1.33)
Model 3^e^	1.00 (reference)	0.75 (0.60, 0.95)	0.73 (0.57, 0.94)	0.98 (0.74, 1.30)
Model 4^f^	1.00 (reference)	0.74 (0.59, 0.94)	0.71 (0.55, 0.92)	0.94 (0.71, 1.25)

a *IMT, intima median thickness; BMI, body mass index*.

b *Adjusted for age, sex*.

c *Odds ratio (95% confidence interval) (all such values)*.

d *Adjusted for age, sex, BMI*.

e *Additional adjusted for smoking status, drinking status, education level, physical activity, employment status, household income, total energy intake, marital status, metabolic syndrome, dietary patterns, and visiting friends*.

f *Additional adjusted for onion intake*.

In stratified analyses, the associations between raw garlic consumption and thickened cIMT were generally similar across all subgroups (Table 4). It did not materially alter the association. For the unmarried people (only 2.93% of all participants), the logistic regression model was not well-fitted and there was no result. All interactions were not statistically significant (*P* for interaction > 0.05). Similar results were observed when we adjusted for three dietary patterns separately in sensitivity analysis (data not shown).

**Table 4:** Multivariate adjusted odds ratios for thickened cIMT according to frequency of raw garlic consumption among Chinese adults stratified by major covariates^a^.

	**Frequency of raw garlic consumption**				***P* for interaction^c^**
	**<1 time/week**	**1 time/week**	**2–3 times/week**	**≥4 times/week**	
Age (years)					0.28
<65	1.00 (reference)	0.68 (0.53, 0.88)^b^	0.70 (0.53, 0.93)	0.83 (0.61, 1.13)	
≥65	1.00 (reference)	1.20 (0.64, 2.26)	0.66 (0.33, 1.29)	1.76 (0.78, 4.05)	
Sex					0.67
Men	1.00 (reference)	0.69 (0.50, 0.94)	0.63 (0.45, 0.88)	0.91 (0.63, 1.31)	
Women	1.00 (reference)	0.84 (0.58, 1.22)	0.80 (0.53, 1.20)	0.89 (0.55, 1.43)	
Smoking status					0.69
Smoker	1.00 (reference)	0.55 (0.33, 0.94)	0.66 (0.38, 1.14)	0.61 (0.33, 1.21)	
Ex-smoker	1.00 (reference)	1.01 (0.42, 2.46)	0.70 (0.29, 1.72)	1.09 (0.41, 2.94)	
Non-smoker	1.00 (reference)	0.85 (0.63, 1.14)	0.76 (0.54, 1.06)	1.10 (0.75, 1.60)	
Drinker status					1.00
Everyday	1.00 (reference)	0.79 (0.33, 1.90)	0.71 (0.29, 1.74)	0.76 (0.30, 1.89)	
Sometime	1.00 (reference)	0.67 (0.48, 0.94)	0.68 (0.41, 0.97)	0.92 (0.61, 1.37)	
Ex-drinker	1.00 (reference)	1.65 (0.73, 3.78)	0.72 (0.28, 1.80)	1.36 (0.48, 3.81)	
Non-drinker	1.00 (reference)	0.75 (0.49, 1.16)	0.67 (0.41, 1.09)	0.90 (0.50, 1.61)	
Physical activity (METs × hour/week)					0.38
≥23	1.00 (reference)	0.60 (0.45, 0.81)	0.63 (0.46, 0.87)	0.66 (0.45, 0.96)	
<23	1.00 (reference)	1.19 (0.80, 1.77)	0.93 (0.61, 1.41)	1.63 (1.04, 2.58)	
Education level					0.77
≥College graduate	1.00 (reference)	0.92 (0.61, 1.41)	0.99 (0.63, 1.56)	0.82 (0.49, 1.37)	
< College graduate	1.00 (reference)	0.67 (0.50, 0.90)	0.58 (0.42, 0.79)	0.99 (0.70, 1.40)	
Household income					0.07
≥10,000 Yuan	1.00 (reference)	0.68 (0.46, 1.02)	0.96 (0.63, 1.48)	1.21 (0.75, 1.95)	
<10,000 Yuan	1.00 (reference)	0.80 (0.60, 1.08)	0.61 (0.44, 0.84)	0.82 (0.57, 1.18)	
Marital status					0.50
Married	1.00 (reference)	0.74 (0.58, 0.94)	0.72 (0.55, 0.92)	0.94 (0.71, 1.25)	
Unmarried	1.00 (reference)	-	-	-	
MS					0.57
Yes	1.00 (reference)	0.72 (0.51, 1.02)	0.72 (0.50, 1.03)	0.88 (0.58, 1.35)	
No	1.00 (reference)	0.79 (0.57, 1.09)	0.71 (0.49, 1.01)	1.04 (0.71, 1.53)	

a *BMI, body mass index; IMT, intima median thickness; METs, metabolic equivalents; MS, metabolic syndrome*.

b *Obtained by using multivariable logistic regression model. Adjusted for age, sex, BMI, smoking status, drinking status, education level, physical activity, employment status, household income, total energy intake, marital status, metabolic syndrome, dietary patterns, visiting friends, and onion intake*.

c *P for interaction was calculated using likelihood ratio test*.

## Discussion

This is the first study aiming to investigate the association between dietary raw garlic consumption and thickened cIMT among a large-scale general population. In the study, we adjusted for a large number of confounding factors. First, existing evidence has shown that cIMT was related to age, sex and BMI (30–32). We adjusted for these three variables in our study, and found that light-to-moderate raw garlic consumption was inversely associated with thickened cIMT, and the association disappeared at greater raw garlic consumption. Second, given that socio-demographic factors, lifestyle factors, nutritional status, and chronic diseases can also influence the association between cIMT and raw garlic consumption, (33–35) we adjusted for factors including smoking status, drinking status, education levels, employment status, household income, physical activity, total energy intake, dietary patterns, marital status, MS and visiting friends. Then, we observed similar results after adjustments for these confounding factors. Finally, as allium vegetables, there are some similar compositions in onion and garlic (36). Both of them are in high content of sulfur-containing compounds, which are all related to high antioxidant activity and effective in the management of cardiovascular diseases (37). Meanwhile, we analyzed the data and found that onion intake was negatively correlated with IMT thickening (*P*_for trend_ <0.05). Thus, we further adjusted onion intake in model 4. Then there was a more obvious association between raw garlic consumption frequency and thickened cIMT. It also indicated that light-to- moderate (1 time/week-2–3 times/week) raw garlic consumption was associated with a lower prevalence of thickened cIMT. However, the association disappeared in group of people who consumed raw garlic ≥4 times/week. The sample size was minimized after stratifying these variables, which may lead to the non-statistical significance of the results in some subgroup analysis. However, the findings and overall trend were similar among all subgroups and were robust to sensitivity analysis.

Several randomized clinical trials have been carried out to assess the effects of garlic preparation on cIMT (20, 21, 38, 39). A randomized, placebo-controlled clinical trial conducted in 56 patients with coronary artery disease found that the mean cIMT was reduced after 3 months garlic powder tablets treatment (1,200 μg allicin/tab twice daily) (21). Meanwhile, Orekhov, A.N. et al. also demonstrated that 2-year treatment with garlic powder pills (150 mg twice daily) delayed atherosclerosis progression as measured using cIMT in men with early carotid atherosclerosis (20). Moreover, a previous study conducted in postmenopausal women suggested that after 12 months of treatment with a phytoestrogen-rich herbal preparation (500 mg daily), including tannins from grape seeds, green tea leaves, hop cone powder, and garlic powder, the mean cIMT progression was significantly lower than in placebo group (39). In contrast, another clinical trial study that focused on perimenopausal women who had 24 months of treatment with a phytoestrogen-rich herbal preparation (500 mg three times daily), including garlic powder, indicated there was no statistically significant difference between the two groups in cIMT (38). Different participants, study durations, and compositions and quantities of sulfur components of different garlic preparations used in various studies may account for inconsistent findings.

According to Chinese eating habits, fresh raw garlic cloves are usually chewed, chopped or crushed directly. Then, alliin, the main active substance in garlic, will get converted into allicin with enzyme alliinase activation. Allicin is liable for most of the pharmacological activity such as hydroxyl radicals scavenging and inhibition of superoxide production and it is metabolized immediately under enzyme-inhibiting gastrointestinal conditions to allyl methane thiosulfinates, methyl methanethiosulfonate and other molecules (40–42). The “allicin bioavailability” is used to represent such processes. Allicin could alleviate atherosclerosis process by inhibiting NO formation (40). Studies showed that high NO concentration resulted in peroxynitrite formation, which could initiate LDL oxidation and then aggravate atherosclerosis process (43, 44). Horev-Azaria et al. found allicin and its derivatives in preventing reactive oxygen species damage by up-regulating the phase II detoxifying enzymes and increasing the cellular glutathione level (45). Li et al. found that allicin inhibits the P38 and JNK pathways and the expression of NF-κB in rats, which also explains the potential anti-inflammatory mechanisms of allicin (46). It is well-established that oxidative stress and inflammation are considered to led to atherosclerosis (8, 9). Furthermore, allicin's role in regulating lipid metabolism has been demonstrated: the allicin-induced upregulation of ABCA1 promotes cholesterol efflux and reduces lipid accumulation via PPARγ/LXRα signaling in THP 1 macrophage-derived foam cells, which plays an important role in reducing the risk of atherosclerosis (47). Additionally, selenium and phytoestrogen in garlic also directly inhibit oxidative stress, modulating inflammation, suppressing endothelial dysfunction, and protecting vascular cells against apoptosis and calcification (38, 48).

Interestingly, the current results suggested that when adults consumed raw garlic ≥4 times/week, the inverse association disappeared. There was no research on raw garlic about dose levels of effective functions. A few reports highlight some of the adverse and toxic effects of higher garlic homogenate (49–51). One experiment in rats found that, after 30 days of eating fresh garlic homogenate at a dose of 250 mg/kg/day, superoxide dismutase (SOD) increased significantly, and reduced thiobarbituric reactive substances (TBARS), a marker of lipid peroxidation. However, 500 and 1,000 mg/kg/day doses significantly reduced endogenous antioxidants (catalase and SOD) without altering TBARS (50). Another animal study also showed that a high dose of garlic may elicit pro-oxidant conditions due to alterations in cell structure and function (50, 51). Moreover, it has been demonstrated that sulphoxides present in garlic extract can undergo exchange reactions with the tritable SH-groups of enzymes and other proteins in the body spontaneously at physiological pH and temperature, inhibiting anti-oxidative activity of these enzymes (49). Evidence above all suggested that high dose of raw garlic consumption may offset the beneficial effect caused by itself. All in all, further research needs to identify optimal garlic consumption and its exact mechanisms.

The strengths of our study include a large sample size and the adjustment of potential confounding factors, such as socio-demographic, lifestyle and clinically relevant variables. However, some limitations of this research must be listed. First, the raw garlic consumption was from self-reported information, which may bring recall bias. Then, although our FFQ was validated to have a reasonably high validity, measurement errors may still exist in self-reported diet and covariates, which usually attenuate true associations (52). Second, we could not infer the causality between raw garlic consumption and cIMT due to its cross-sectional design. Therefore, prospective studies should be conducted to confirm the association. Finally, despite we adjusted for abundant confounding factors, there will be other residual factors we may not fully capture.

## Conclusions

This is the first study to investigate the association between raw garlic consumption frequency and thickened cIMT. According to our results, light-to-moderate (1 time/week-2–3 times/week) raw garlic consumption was inversely associated with thickened cIMT. More prospective studies with long-term follow-up will be necessary to confirm the preliminary findings of the current study.

## Data Availability Statement

The data of this study (in de-identified form) can be requested from the corresponding authors. (E-mail: nkj0809@gmail.com or sz7ytfl@126.com).

## Ethics Statement

The studies involving human participants were reviewed and approved by Medical Ethics Committee of the Tianjin Medical University with the reference number of TMUhMEC 201430. The patients/participants provided their written informed consent to participate in this study. Written informed consent was obtained from the individual(s) for the publication of any potentially identifiable images or data included in this article.

## Author Contributions

YL and GM analyzed data and wrote the paper. GM, YL, QZ, LL, HW, YG, SZ, TZ, XW, SS, MZ, QJ, and KS conducted the research. KN and FT designed the research and had primary responsibility for final content. All authors read and approved the final manuscript.

## Conflict of Interest

The authors declare that the research was conducted in the absence of any commercial or financial relationships that could be construed as a potential conflict of interest.

## Abbreviations

BMI: body mass index
BP: blood pressure
CCA: common carotid artery
CI: confidence interval
cIMT: carotid intima-media thickness
CVD: cardiovascular disease
FBG: fasting blood glucose
FFQ: food frequency questionnaire
HDL-C: high-density lipoprotein cholesterol
LDL-C: low-density lipoprotein cholesterol
METs: metabolic equivalents
MS: metabolic syndrome
OR: odds ratio
PA: physical activity
SOD: superoxide dismutase
TBARS: thiobarbituric acid reactive substances
TC: total cholesterol
TCLSIH: Tianjin Chronic Low-grade Systemic Inflammation and Health
TG: triglycerides
WC: waist circumference
WDRs: weighed diet records.
