# Supplementary material for: Light-To-Moderate Raw Garlic Consumption Frequency Is Inversely Associated With Thickened Carotid Intima-Media Thickness: A Population-Based Study
Source: Front Nutr. 2021 Mar 31;8:648821. doi: 10.3389/fnut.2021.648821 (PMC8044311; doi:10.3389/fnut.2021.648821)
Supplement: Supplementary file 1 [file Table_1.DOCX]

| **Supplementary Table 1.** Factor loadings for food items derived from factor analysis (principal component analysis) * | | | | | |
| --- | --- | --- | --- | --- | --- |
| Health dietary pattern | | Sweets dietary pattern | | Animal foods dietary pattern | |
| Food items | Factor loadings | Food items | Factor loadings | Food items | Factor loadings |
| cucumber | 0.59 | western-style pastry, cakes | 0.59 | animal offal (except for animal liver) | 0.62 |
| pumpkin, carrot | 0.56 | pineapple | 0.58 | animal blood | 0.57 |
| celery | 0.55 | strawberry, kiwi fruit, persimmon | 0.57 | animal liver | 0.54 |
| tomato (including the ketchup) | 0.54 | ice cream | 0.55 | preserved egg | 0.53 |
| Chinese cabbage | 0.54 | Chinese cakes | 0.54 | freshwater fish | 0.49 |
| mushroom | 0.53 | grape | 0.52 | pork skin | 0.49 |
| soya bean products | 0.52 | sweets, candied fruits | 0.50 | seafood (shellfish, squid, shrimp) | 0.49 |
| green vegetable | 0.50 | peach | 0.48 | sausage | 0.49 |
| radish (expect for carrot) | 0.49 | cookies | 0.47 | sea fish | 0.48 |
| eggplant | 0.48 | banana | 0.43 | wonton | 0.41 |
| other types of beans | 0.48 | watermelon | 0.40 | instant noodle | 0.40 |
| apple | 0.47 | pear | 0.36 | poultry | 0.39 |
| potato (except for sweet potato) | 0.46 | fruit juice, vegetable juice | 0.36 | strong liquor | 0.38 |
| bell peppers | 0.46 | carbonated beverage | 0.36 | meat | 0.38 |
| Chinese watermelon | 0.46 | other kinds of fruit | 0.33 | leek | 0.35 |
| raw vegetables (except for tomato) | 0.46 | Chinese sauerkraut | 0.32 | Chinese sauerkraut | 0.33 |
| garlic | 0.46 | coffee | 0.32 | low-alcohol liquor | 0.33 |
| pear | 0.45 | nuts | 0.32 | salted eggs | 0.32 |
| * Only factor loading greater than \|0.30\| | | | | | |
